# β‐1,4‐Galactosyltransferase 1 protects against cerebral ischemia injury in mice by suppressing ferroptosis via the TAZ/Nrf2/HO‐1 signaling pathway

**DOI:** 10.1111/cns.70030

**Published:** 2024-09-04

**Authors:** Yao Ma, Chang Liu, Lili Ren, Jiachen Li, Yunhao Xu, Jia Liang, Peng Wang

**Affiliations:** ^1^ Department of Neurobiology, School of Basic Medicine Jinzhou Medical University Jinzhou Liaoning China; ^2^ Institute of Life Science, Jinzhou Medical University Jinzhou Liaoning China; ^3^ Liaoning Provincial Key Laboratory of Neurodegenerative Diseases Jinzhou Medical University Jinzhou Liaoning China

**Keywords:** B4galt1, ferroptosis, ischemic stroke, Nrf2, TAZ

## Abstract

**Background:**

Ischemic stroke leads a primary cause of mortality in human diseases, with a high disability rate worldwide. This study aims to investigate the function of β‐1,4‐galactosyltransferase 1 (B4galt1) in mouse brain ischemia/reperfusion (I/R) injury.

**Methods:**

Recombinant human B4galt1 (rh‐B4galt1) was intranasally administered to the mice model of middle cerebral artery occlusion (MCAO)/reperfusion. In this study, the impact of rh‐B4galt1 on cerebral injury assessed using multiple methods, including the neurological disability status scale, 2,3,5‐triphenyltetrazolium chloride (TTC), Nissl and TUNEL staining. This study utilized laser speckle Doppler flowmeter to monitor the cerebral blood flow. Western blotting was performed to assess the protein expression levels, and fluorescence‐labeled dihydroethidium method was performed to determine the superoxide anion generation. Assay kits were used for the measurement of iron, malondialdehyde (MDA) and glutathione (GSH) levels.

**Results:**

We demonstrated that rh‐B4galt1 markedly improved neurological function, reduced cerebral infarct volume and preserved the completeness of blood–brain barrier (BBB) for preventing damage. These findings further illustrated that rh‐B4galt1 alleviated oxidative stress, lipid peroxidation, as well as iron deposition induced by I/R. The vital role of ferroptosis was proved in brain injury. Furthermore, the rh‐B4galt1 could increase the levels of TAZ, Nrf2 and HO‐1 after I/R. And TAZ‐siRNA and ML385 reversed the neuroprotective effects of rh‐B4galt1.

**Conclusions:**

The results indicated that rh‐B4galt1 implements neuroprotective effects by modulating ferroptosis, primarily via upregulating TAZ/Nrf2/HO‐1 pathway. Thus, B4galt1 could be seen as a promising novel objective for ischemic stroke therapy.

## Graphical abstract

Ferroptosis occurs during cerebral ischemia/reperfusion, resulting in cerebral injury. Rh‐B4galt1 exerts neuroprotective effects by regulating ischemia‐induced ferroptosis, which is mainly dependent on the heightening of the TAZ/Nrf2/HO‐1 pathway. Thus, B4galt1 may be considered a novel potential therapeutic target for ischemic stroke treatment.

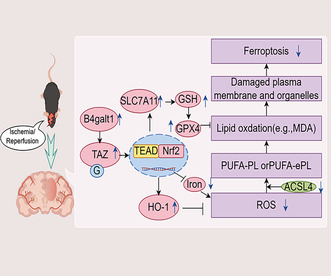

## 1 INTRODUCTION

Ischemic stroke ranks as a primary reason of death in human disorders, with a high disability rate across the globe. Although some advances in treatment, finding suitable treatment methods for cerebral ischemic injury remains a challenge.^1^ The pathology in cerebral ischemic injury is complicated, involving factors such as oxidative stress, cell apoptosis, and inflammation, finally leading neuronal death.^2^

After ischemic stroke, brain glycosylation level is vibrantly restricted, markedly impacting the blood–brain barrier (BBB) disruption.^3^ Moreover, recent researches have demonstrated that drugs targeting protein glycosylation mitigate brain damage in experimental stroke model.^4^, ^5^ Therefore, the significance upon protein glycosylation within prognosis of ischemic stroke directs the advancement of innovative treatments for this condition.

Glycosyltransferases hold essential state in glycosylation process, determining the proteins and positions that undergo glycosylation. Our research findings have indicated that β‐1,3‐galactosyltransferase 2 plays a significant role in cerebral ischemic injury.^6^, ^7^, ^8^ β‐1,4‐Galactosyltransferase 1 (B4galt1) adds galactose toward N‐glycans to form type 2 LacNAc units. Although few studies have analyzed the function of B4galt1, in an analysis of the dataset GSE104036, B4galt1 was identified as a gene that is up‐regulated following acute ischemic stroke.^3^ So, in this study, the function of B4galt1 was investigated through the intranasal administration of recombinant human B4galt1 (rh‐B4galt1). Intranasal delivery is gaining popularity for central nervous system research purposes.^9^, ^10^

Ferroptosis is controlled by glutathione peroxidase 4 (GPX4), as well as radical‐trapping antioxidants.^11^, ^12^ In recent studies, ACSL4 contributed to ischemic neuronal death by inducing neuronal ferroptosis, which is demonstrated to be associated with several brain disorders. Ischemic stroke was included in them.^13^, ^14^, ^15^, ^16^ Currently, inhibiting ferroptosis has been seen as a novel therapeutic method in managing ischemic stroke. Nevertheless, specific mechanism through which rh‐B4galt1 modulates ischemia‐induced ferroptosis remains unclear.

This research was conducted for exploring the effects of rh‐B4galt1 on ischemic brain damage and elucidating the mechanism through which rh‐B4galt1 inhibited ferroptosis in ischemia/reperfusion (I/R) model of mouse. The research was looking forward to provide new potential targets for treating ischemic stroke.

## 2 MATERIALS AND METHODS

### 2.1 Experimental animals

Male Kunming mice (2–3 months old, weighing 20–25 g) were purchased from the Experimental Animal Center of Jinzhou Medical University and accommodated in thermostatically regulated rooms carrying 12 h light/dark cycles. They were free to get essential nutrients throughout the experiment. A total of 372 male mice were used in the experiment. Only male mice were used due to potential effects of sex hormones. The experimental protocols were approved by the Animal Care and Use Committee of Jinzhou Medical University. Treatment assignments were randomized for all mice.

### 2.2 Focal cerebral ischemia model preparation

Middle cerebral artery occlusion (MCAO) was used to provoke cerebral ischemic damage, following established protocols.^17^, ^18^ In short, mice were sedated using 2% isoflurane in N_2_O and O_2_ (7:3) blend. During surgery, the right common carotid artery was in a state of bursting, as well the ipsilateral internal carotid artery. The surgical nylon monofilament (180 μm diameter) was utilized for inducing focal cerebral ischemia for MCAO. One hour later, reperfusion was initiated by removing the monofilament. The sham operation mice were carried out with the same procedure, but with no nylon monofilaments. Homeothermic warming mat was carefully utilized to remain room temperature. The efficacy on cerebral ischemia model was assessed through monitoring the directional circling behavior of the mice toward the non‐ischemic side (left) and subsequently proved through TTC staining.

### 2.3 Pharmaceutical supervision and management

Intranasal administration as described earlier: mice were supine and given three doses of rh‐B4galt1 (Abnova), as doses were 0.02, 0.06 and 0.18 μg/kg.^19^, ^20^ After MCAO, a total of 10 μL of rh‐B4galt1 dissolved in PBS was executed intranasally within 10 min, and then the mice were supine for 5 min for drug absorption. Mice in Deferoxamine (DFO, 100 mg/kg, i.p.) group were given DFO. ML385, a Nrf2 inhibitor (30 mg/kg), was delivered by intraperitoneal administration after MCAO.

### 2.4 Intracerebroventricular injection

Pre‐designed TAZ‐targeted small interfering RNA (siRNA) was administered via intraventricular injection 24 h prior to MCAO. TAZ siRNA was delivered to tissue using Lipofectamine RNAiMAX Transfection Reagent (Invitrogen, Carlsbad, CA, USA). The mice were fixed in a stereotactic device after anesthesia with pentobarbital sodium. TAZ siRNA of 2 μL, 300 pmol/μL was administered in ipsilateral ventricle at a rate of 0.3 μL/min (0.6 mm posterior to bregma, 1.5 mm lateral to bregma, and 1.7 mm ventral to the brain surface). After injection, keep the needle still for 5 min to prevent leakage and then slowly remove it within 5 min.

### 2.5 Measurement upon neurological deficits

The Neurological Deficit Scoring System (NDSS) was carried out for evaluating neurological deficits, ranging from 0 to 10.^21^ Neurological assessments were conducted in a blinded way.

### 2.6 Assessment upon infarct volume

After perfusion with physiological saline through the heart, the mouse brain was sliced coronally into sections of 1.0 mm thickness in a very short time. These gotten sections of the brain were incubated in 0.5% TTC solution at room temperature for ~10 min. The photomicrographs of stained slices were recorded and measured through ImageJ software. The infarction size is represented as a percentage by deducting the non‐infarcted region of ipsilateral from that of contralateral hemisphere and dividing by the contralateral hemisphere area.

### 2.7 Nissl staining

As described earlier, following blood flow, the murine brain tissue was fixed. The dehydrated brains were divided to 20 μm slices, consecutively immersed in 20% and 30% sucrose solutions, and colored with 0.04% cresyl violet dissolved within acetic acid buffer in 1 h.

### 2.8 TUNEL staining

The TUNEL assay kit was carried out for assessing cell apoptosis. Briefly, these sections of brain were cultured in permeabilization for 5 min, following with a 1‐h dark culturing at room temperature with TUNEL reagents. Neurons and neural nuclei were visualized using NeuN (1:3000, Chemicon, Temecula, CA) and 4′,6‐diamidino‐2‐phenylindole (DAPI) stains, respectively. At last, the fluorescence microscope was carried out for getting images.

### 2.9 Evaluation of BBB disruption

Fluorescent tracer FITC‐conjugated glucan was controlled intravenously through the tail vein. Cycle for 1 h, 4% paraformaldehyde was perfused through the heart. The 30 μm thick coronary brain slices were processed and FITC was directly detected using fluorescence. The cerebral volume of FITC glucan leakage upon six equidistant cerebral slices containing the MCA region was calculated.

### 2.10 Cerebral blood flow monitoring

After 24 h of reperfusion, we monitored the cerebral blood flow (CBF) of each group using a laser speckle Doppler flowmeter (RFLSI III, RWD Life Science Co, Shenzhen, China). The images and data were analyzed using RFLSI analysis software (RWD Life Science).

### 2.11 Immunofluorescence

The 20 μm thick cerebral slices were blocked in 5% normal goat serum for 1 h, then incubated with primary antibodies for 12 h, mainly including B4galt1 (1:500, Affinity), GFAP (1:500, Invitrogen), NeuN (1:300, Invitrogen), Occludin (1:500, Invitrogen), Claudin‐5 (1:200, Invitrogen), and CD31 (1:100, Abcam). Then incubated at room temperature with secondary antibody (1:4000, Invitrogen) for ~2 h. Slices were reverse stained with DAPI.

### 2.12 Reactive oxygen species (ROS) production

These mouse brains were extracted, then, fix the brain back in 4% polyaldehyde. After continuous soaking, the brain was cut to be 20 μm thick slices. The production of superoxide anion was measured through fluorescent‐labeled dihydroethidium staining.

### 2.13 Measurement of MDA, GSH, and iron

The supernatant was collected from the homogenized ischemic tissue. The levels of glutathione (GSH) and malondialdehyde (MDA) were evaluated through detection kits. The iron level was evaluated a tissue iron analysis kit.

### 2.14 Western blotting

Protein samples were extracted from the ischemic hemisphere. The tissue was homogenized in radioimmunoprecipitation assay (RIPA) buffer (Beyotime, China) using sonication. The protein concentration was determined using a BCA protein assay kit (Beyotime, China). Equal amounts of protein (30 μg) were separated by 10% SDS‐PAGE. The antibodies used in this study were B4galt1 (1:500, Affinity), GPX4 (1:800, ABclonal), SLC7A11 (1:800, ABclonal), ACSL4 (1:500, ABclonal), TAZ (1:1000, ABclonal), nuclear factor erythroid 2‐related factor 2 (Nrf2, 1:1000, ABclonal), heme oxygenase‐1 (HO‐1, 1:1000, ABclonal), GAPDH (1:7500, Sigma) and corresponding secondary antibodies (1:5000, Invitrogen). The membrane was incubated with the primary antibody overnight and with the secondary antibody for 2 h. Signal detection was carried out by Enhanced Chemiluminescence (ECL) assay kit. The protein levels were detected and standardized to the internal reference GAPDH using densitometry.

### 2.15 Statistical analysis

All data are shown as mean ± SD. Statistical analyses were performed using GraphPad Prism version 7. Normality was determined by Shapiro–Wilk test. For normally distributed data, statistical differences were evaluated with Student's *t‐*test between two groups or by one‐way analysis of variance (ANOVA) followed by Bonferroni/Dunn post hoc test among more than two groups. Comparisons for non‐normally distributed data were performed using nonparametric Mann–Whitney test or Kruskal–Wallis test. *p* < 0.05 was defined as statistically significant.

## 3 RESULTS

### 3.1 The elevation of B4galt1 following focal cerebral ischemia

**FIGURE 1:**
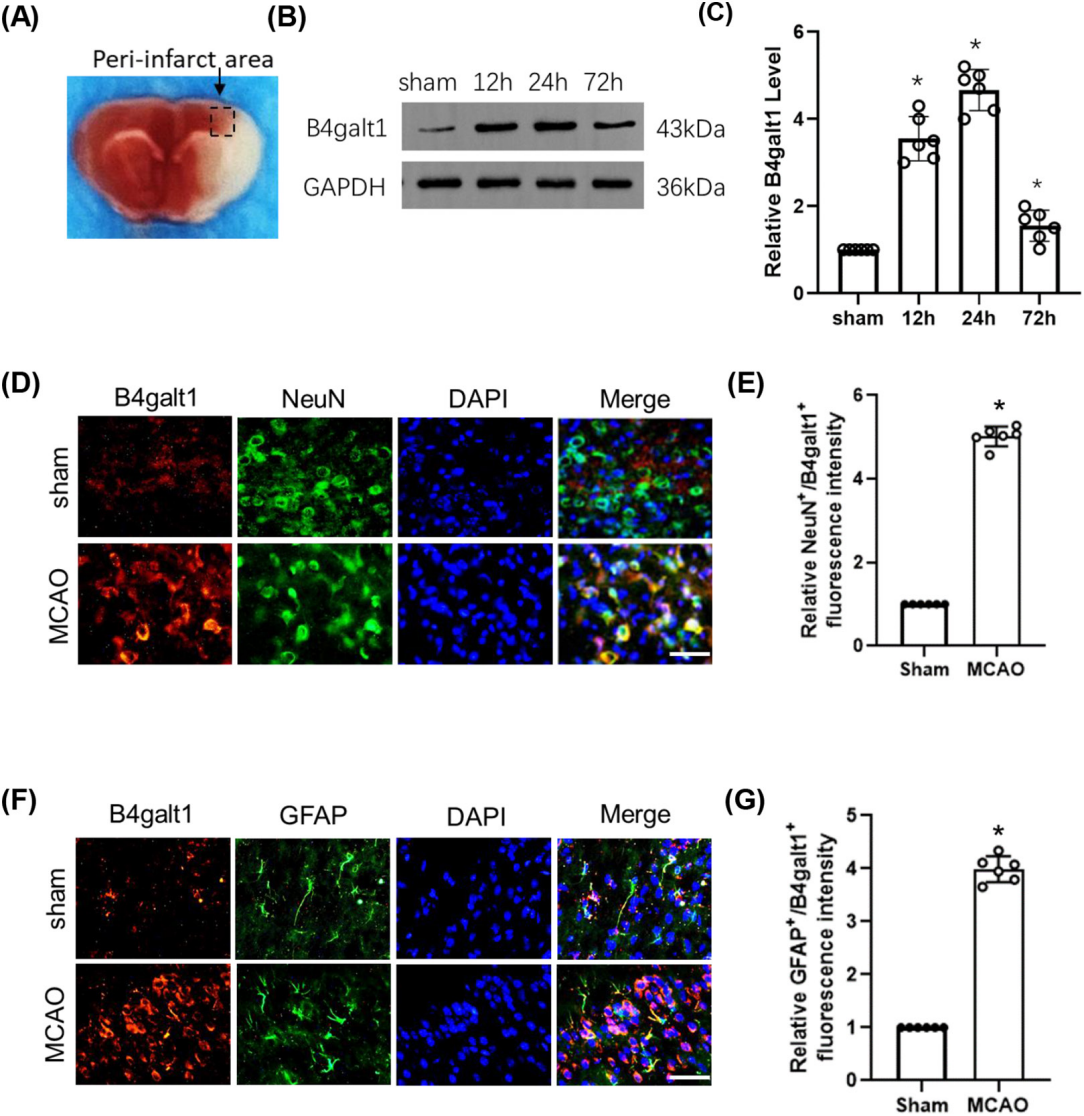
The levels of B4galt1 at 12 h, 24 h, 72 h after I/R. (A) Representative image of the peri‐infarct penumbra. (B) Representative pictures of Western blotting data in the peri‐infarct penumbra of mice. (C) Quantitative analysis of the protein level of B4galt1. (D, F) Representative double immunostaining of B4galt1 (red) with NeuN (a neuronal marker, green) and GFAP (an astrocyte glial marker, green) from ischemic penumbra of brain tissue after MCAO surgery. (E, G) Quantification of NeuN^+^/B4galt1^+^ and GFAP^+^/B4galt1^+^ fluorescence intensity was quantified using ImageJ. Scale bars, 50 μm. *p < 0.05 versus Sham group. Data are expressed as mean ± SD, *n* = 6 for each group.

To detect the expression level of B4galt1 following focal cerebral ischemia, we subjected mice to MCAO for 1 h, followed by reperfusion for 12, 24, and 72 h. These findings indicated that B4galt1 in the peri‐infarct penumbra of mice was significantly up‐regulated after reperfusion and reached its peak at 24 h compared with sham surgery group (Figure 1A–C). Immunofluorescence staining was conducted to identify the cell types expressing B4galt1 in the brain. Particularly, B4galt1 expression was predominantly observed in neurons and astrocytes (Figure 1D–G).

### 3.2 The effects of rh‐B4galt1 administration on cerebral ischemic injury

**FIGURE 2:**
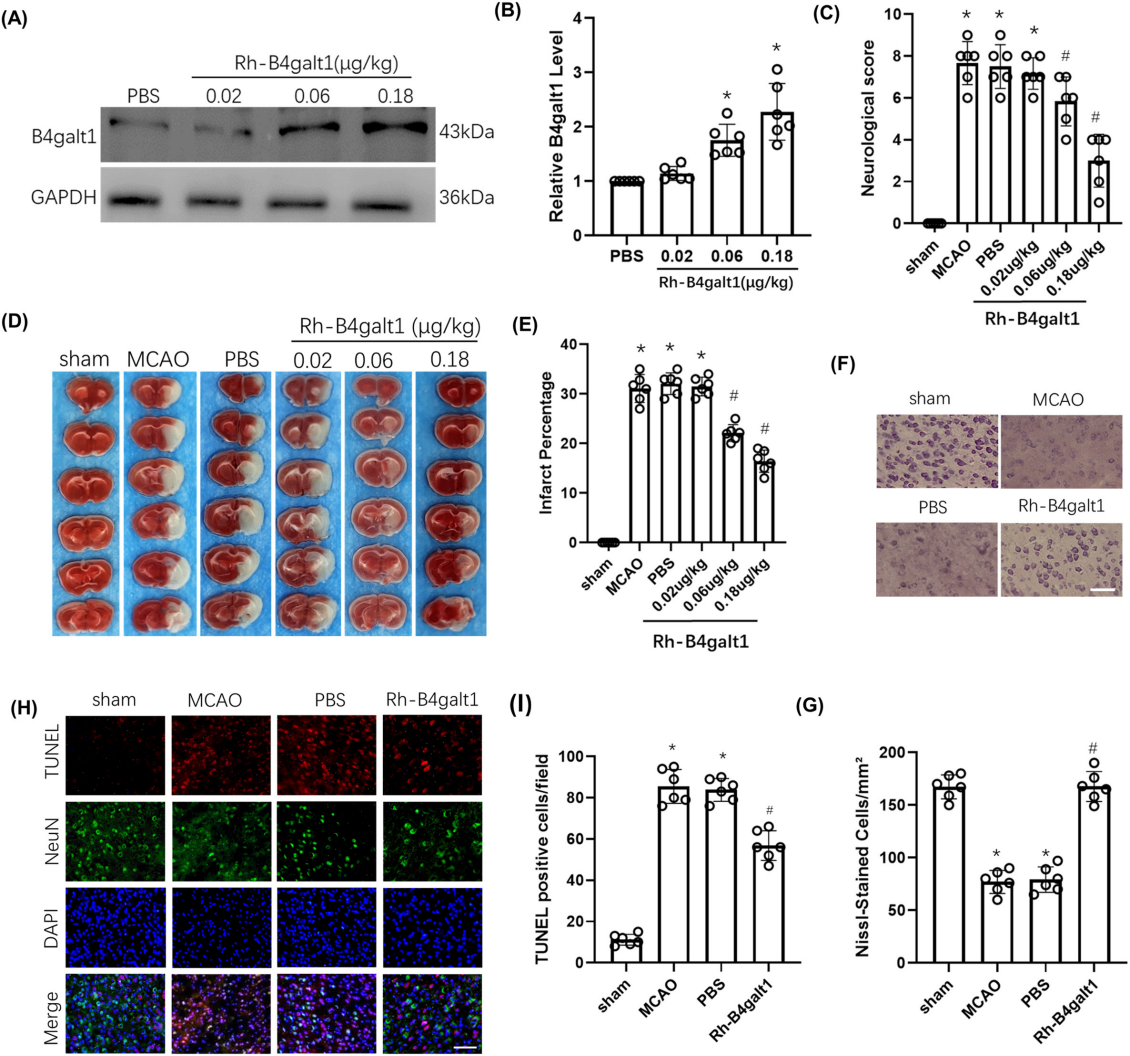
Effects of intranasal administration of rh‐B4galt1 on brain damage after I/R. (A, B) Representative Western blotting images and quantitative analysis of the levels of B4galt1. *p < 0.05 versus PBS group. Data are expressed as mean ± SD, *n* = 6 for each group. (C) Neurological deficit scores. (D) Representative images of TTC staining illustrating cerebral infarct volumes in mice with 1 h MCAO/24 h reperfusion. (E) Quantitative analysis of infarct volumes. (F) Typical images of Nissl‐staining in the penumbra. Scale bar, 100 μm. (G) Quantitative analysis of Nissl‐staining. (H) Typical images of TUNEL staining in the penumbra. (I) Quantification of TUNEL‐positive cells. Scale bar, 50 μm. *p < 0.05 versus Sham group; ^#^ *p* < 0.05 versus MCAO group. Data are expressed as mean ± SD, *n* = 6 for each group.

The optimal dose of rh‐B4galt1 for reducing cerebral ischemic injury was determined by evaluating three different doses: 0.02, 0.06, and 0.18 μg/kg. Intranasal administration of 0.06 and 0.18 μg/kg of exogenous rh‐B4galt1 increased B4galt1 levels (Figure 2A,B). NDSS showed that the MCAO group had worse performance than the sham group. 0.06 and 0.18 μg/kg of rh‐B4galt1 significantly improved neurological function compared with the MCAO group (Figure 2C). And the 0.06 and 0.18 μg/kg rh‐B4galt1 markedly decreased infarct volume than MCAO group from TTC staining (Figure 2D,E). To sum up, intranasal administration of rh‐B4galt1 was protective following cerebral ischemia–reperfusion, with 0.18 μg/kg dose showing the greatest function and being selected for follow‐up experiments.

Nissl staining revealed substantial neuronal loss in the peri‐infarct penumbra following ischemia. After 24 h of reperfusion, rh‐B4galt1 could alleviate this loss (Figure 2F,G). We also used TUNEL staining for confirming the function of rh‐B4galt1. The above findings revealed that there was almost no cell apoptosis in sham group, while the MCAO group and PBS group had more apoptosis in the penumbra. Rh‐B4galt1 can markedly reduce neuronal apoptosis after brain I/R (Figure 2H,I).

### 3.3 The function of Rh‐B4galt1 treatment in BBB and CBF

**FIGURE 3:**
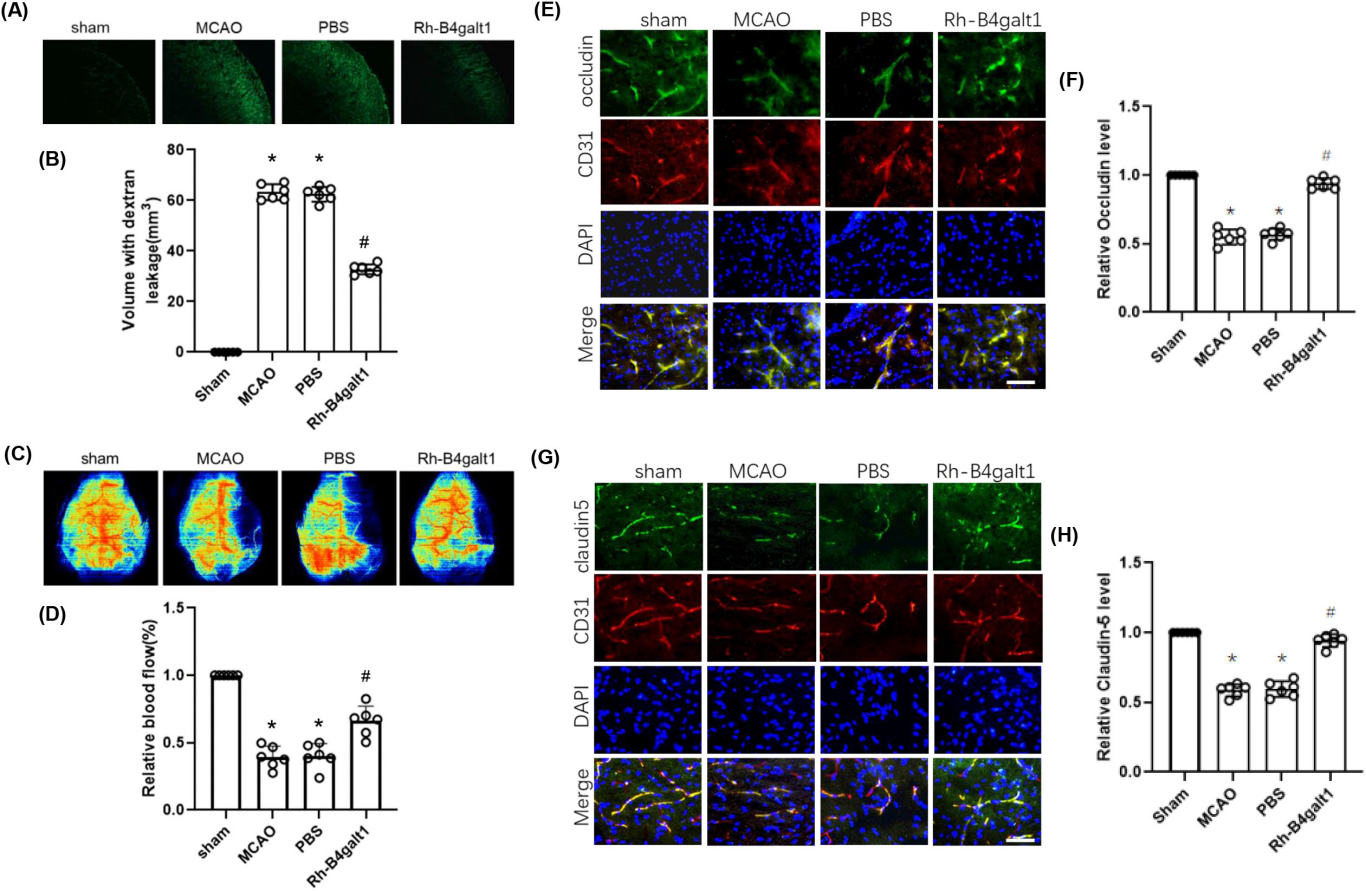
Rh‐B4galt1 reduced BBB leakage after I/R injury. (A) Brain slices showing FITC‐dextran leakage. (B) Quantitative analysis of FITC‐dextran content in ischemic hemisphere. (C) Laser speckle imaging at 24 h after reperfusion. (D) Quantification of relative blood flow. (E) Co‐staining of occludin (green), CD31 (an endothelial cell marker, red) and DAPI (blue) in the penumbra. (F) Quantitative analysis of occludin. (G) Co‐staining of claudin‐5 (green), CD31 (red) and DAPI (blue) in the penumbra. (H) Quantitative analysis of claudin‐5. Scale bar: 50 μm. *p < 0.05 versus Sham group; ^#^ *p* < 0.05 versus MCAO group. Data are expressed as mean ± SD, *n* = 6 for each group.

The results of FITC‐dextran extravasation suggested that under I/R conditions the content of FITC‐dextran significantly increased, while the use of rh‐B4galt1 significantly reduced FITC‐dextran extravasation (Figure 3A,B). After 24 h of reperfusion, the results for monitoring the CBF of each group suggested that rh‐B4galt1 treatment slowed down the decrease in CBF compared with the MCAO group (Figure 3C,D).

Further investigation was conducted on the effect of rh‐B4galt1 treatment on tight junction proteins (TJPs) loss in the cerebral microvasculature. Occludin and claudin‐5 contents were evaluated with immunofluorescence staining. These outcomings revealed that their levels were reduced in ischemic microvasculature, while rh‐B4galt1 treatment restored their levels (Figure 3E–H). In summary, rh‐B4galt1 can inhibit the degradation of TJPs, thereby alleviating ischemia‐induced BBB damage.

### 3.4 Rh‐B4galt1 ameliorated ferroptosis in ischemic mice

**FIGURE 4:**
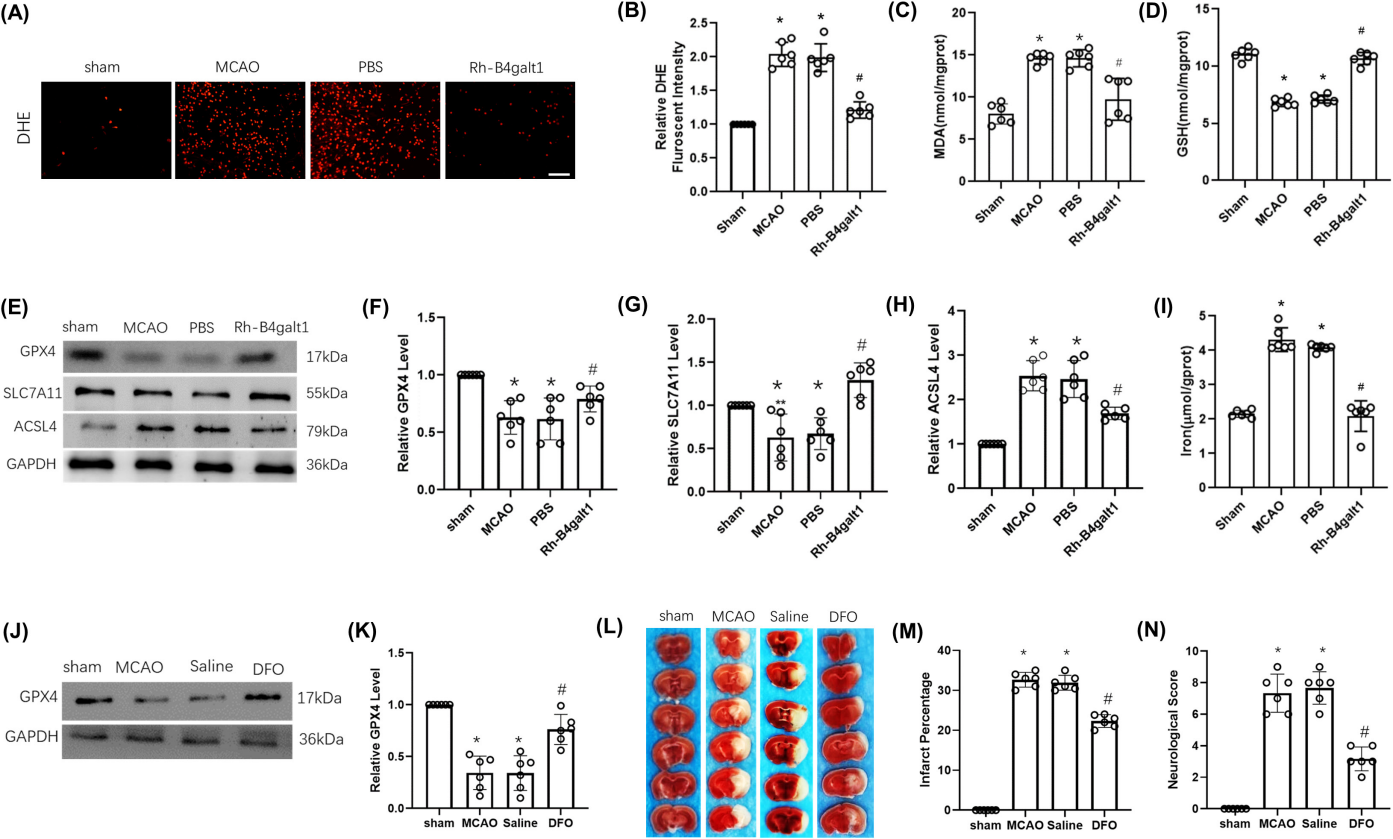
Effects of rh‐B4galt1 on ferroptosis after I/R. (A) Representative images of DHE staining. (B) Quantitative analysis of DHE signals. (C, D) The levels of MDA and GSH were detected by using biochemical kit. (E–H) Representative Western blotting images and quantitative analysis of the levels of the ferroptosis‐related proteins GPX4, SLC7A11, and ACSL4. (I) The iron concentration in the brain tissue. (J) Representative Western blotting images of GPX4. (K) Quantitative analysis of GPX4 level. (L) Representative images of TTC staining illustrating cerebral infarct volumes in mice with 1 h MCAO/24 h reperfusion. (M) Quantitative analysis of infarct volumes. (N) Neurological deficit score analysis of motor behavior. Scale bar, 50 μm. *p < 0.05 versus Sham group; ^#^ *p* < 0.05 versus MCAO group. Data are expressed as mean ± SD, *n* = 6 for each group.

For further exploring the regulatory function of rh‐B4galt1 treatment upon ferroptosis in cerebral ischemic mice, we assessed key markers associated with ferroptosis at 24 h post‐reperfusion. The level of ROS, as measured by DHE staining, was elevated in the penumbra region following I/R injury, but was mitigated by administration of rh‐B4galt1 (Figure 4A,B). I/R also led to an increase in MDA production, which was significantly reduced by the treatment with rh‐B4galt1 (Figure 4C). Furthermore, rh‐B4galt1 treatment markedly elevated the levels of reduced GSH following MCAO (Figure 4D).

In the subsequent experiment, we assessed the levels of various ferroptosis‐related proteins in 1 h of MCAO, following with 24 h of reperfusion using Western blotting. The findings showed the I/R decreased the levels GPX4 and SLC7A11, while ACSL4 expression was raised with 24 h post‐reperfusion (Figure 4E–H). However, compared with the MCAO group, rh‐B4galt1 treatment upregulated GPX4 and SLC7A11, whereas downregulated ACSL4. Additionally, we assayed iron concentration at 24 h of reperfusion. Similarly, iron exhibited notable cytoplasmic accumulation in MCAO and rh‐B4galt1 treatment decreased its concentration in the brain tissue (Figure 4I).

Mice were treated with DFO 24 h prior to MCAO to demonstrate the importance of iron‐mediated cell death inhibition. The expression of GPX4 was shown to decrease during ischemia. Compared with the MCAO group, GPX4 expression was significantly increased in DFO (Figure 4J,K). In addition, DFO treatment decreased cerebral infarction volume and neurological deficit scores in ischemic mice (Figure 4L–N). We propose that rh‐B4galt1 protected the brain from I/R damage through ferroptosis inhibition.

### 3.5 TAZ siRNA and ML385 abolished the protective effects of rh‐B4galt1 on cerebral ischemic injury

**FIGURE 5:**
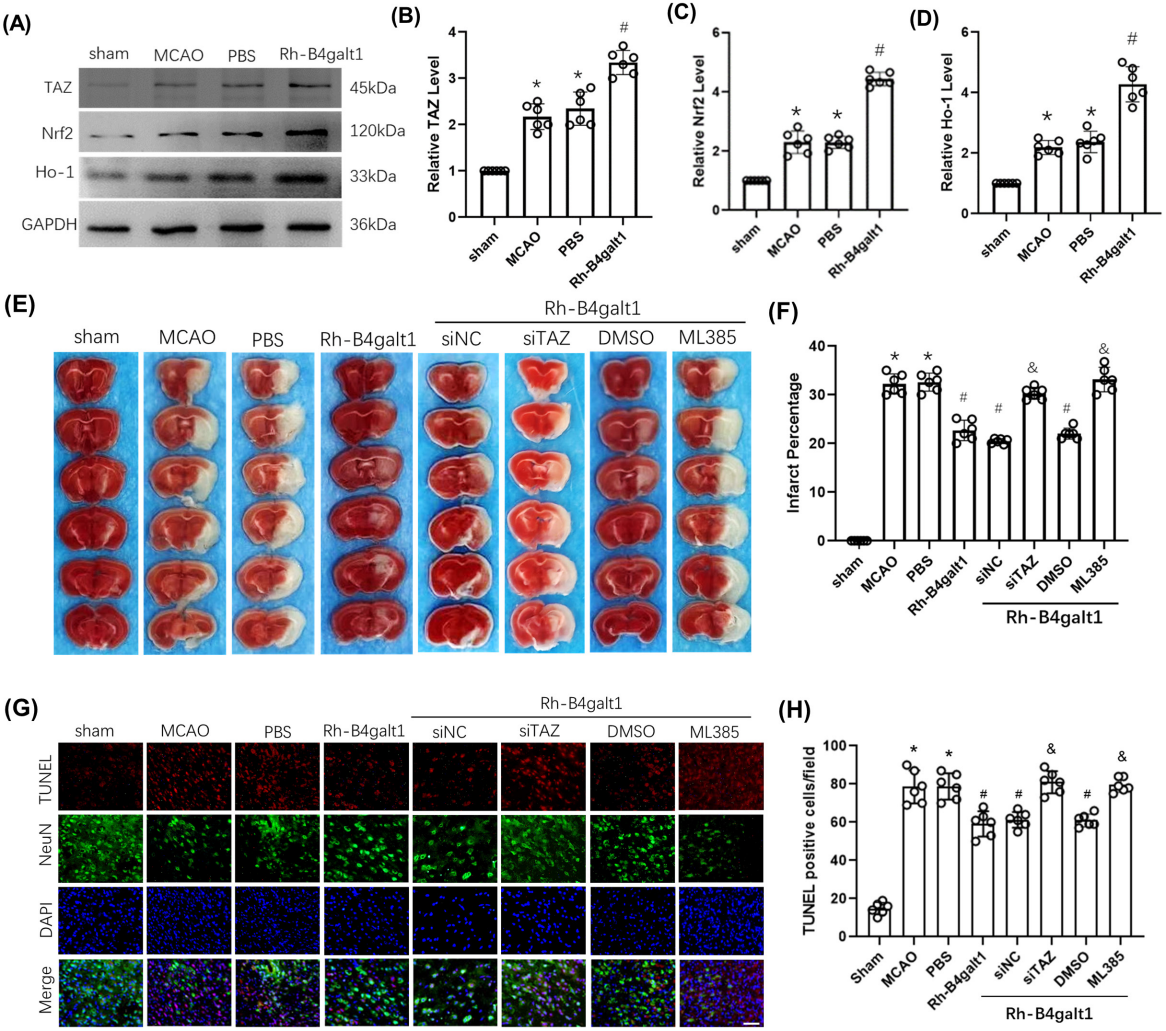
The effects of rh‐B4galt1 on the TAZ/Nrf2/HO‐1 signaling pathway. (A–D) Representative Western blotting images and quantitative analysis of the levels of TAZ, Nrf2, and HO‐1. (E) Representative images of TTC staining illustrating cerebral infarct volumes in mice with 1 h MCAO/24 h reperfusion. (F) Quantitative analysis of infarct volumes. (G) Typical images of TUNEL staining in the penumbra. (H) Quantification of TUNEL‐positive cells. Nuclei were stained with DAPI and neurons were stained with NeuN. Scale bar, 50 μm. *p < 0.05 versus Sham group; ^#^ *p* < 0.05 versus MCAO group; ^&^ *p* < 0.05 versus rh‐B4galt1 group. Data are expressed as mean ± SD, *n* = 6 for each group.

We identified the potential molecular mechanisms of rh‐B4galt1 in cerebral ischemia using Western blotting. The proteins expression, including TAZ, Nrf2, and HO‐1, were increased at 24 h after MCAO (Figure 5A–D). However, the treatment of rh‐B4galt1 further increases their levels than MCAO group. Our results suggested that rh‐B4galt1 could modulate the pathway of TAZ/Nrf2/HO‐1 following cerebral ischemic damage.

Next, TAZ siRNA and ML385 were used as interventions in the pathway for further analysis. The alleviation of cerebral infarction by rh‐B4galt1 treatment was inhibited by TAZ siRNA and ML385 (Figure 5E,F), suggesting they could reverse the protective effects of rh‐B4galt1. In addition, we also used TUNEL staining to further confirm the function of rh‐B4galt1. The suppression effect of rh‐B4galt1 treatment against the upregulation of neuronal apoptosis in peri‐infarct penumbra was reversed by adding TAZ siRNA and ML385 (Figure 5G,H). In a word, the neuroprotective function of rh‐B4galt1 in ischemia‐induced brain damage has relationship with TAZ/Nrf2/HO‐1 pathway.

### 3.6 Rh‐B4galt1 rescued ferroptosis via the TAZ/Nrf2/HO‐1 pathway in mice with cerebral ischemia

**FIGURE 6:**
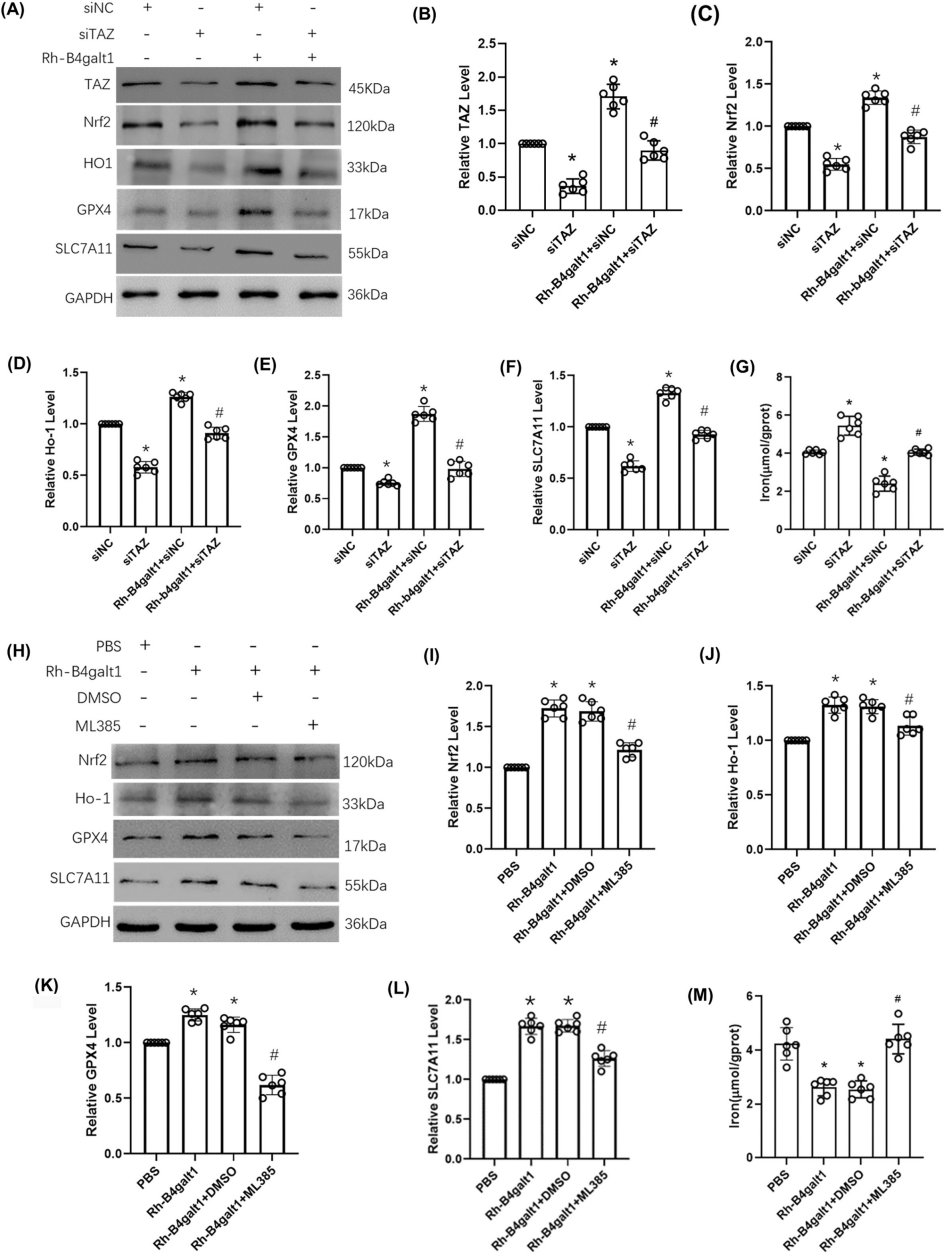
The effects of rh‐B4galt1 on ferroptosis via the TAZ/Nrf2/HO‐1 signaling pathway. (A–F) Representative Western blotting images and quantitative analysis of the levels of TAZ, Nrf2, HO‐1, GPX4 and SLC7A11. (G) The iron concentration in the brain tissue. *p < 0.05 versus siNC group; ^#^ *p* < 0.05 versus Rh‐B4galt1+siNC group. (H–L) Western blotting was carried out to test the expression of Nrf2, HO‐1, GPX4, and SLC7A11. (M) The iron concentration in the brain tissue. *p < 0.05 versus PBS group; ^#^ *p* < 0.05 versus Rh‐B4galt1 group. Data are expressed as mean ± SD, *n* = 6 for each group.

TAZ siRNA and ML385 were delivered along with rh‐B4galt1 to study its mechanism on attenuating ferroptosis. The data from the Western blotting supported that the TAZ, Nrf2, HO‐1, GPX4, and SLC7A11 were markedly reduced with TAZ siRNA, blocking the effects of rh‐B4galt1 (Figure 6A–F). The reduction in brain tissue iron concentration following rh‐B4galt1 treatment was reversed by TAZ siRNA (Figure 6G). As shown in Figure 6H–L, the upregulation of Nrf2, HO‐1, GPX4, and SLC7A11 levels following rh‐B4galt1 treatment was suppressed by ML385. Similarly, the decrease of iron concentration in the brain tissue after rh‐B4galt1 treatment was abolished by ML385 (Figure 6M). Overall, in this study, rh‐B4galt1 has the ability to modulate I/R‐induced ferroptosis via TAZ/Nrf2/HO‐1 pathway.

## 4 DISCUSSION

Increasing evidences suggest the B4galt holds a vital state in central nervous system.^22^, ^23^, ^24^, ^25^ Among them, B4galt1 may be able to regulate apoptosis and autophagy of glioblastoma.^26^ In the present research, we constructed a mouse ischemic stroke model using the MCAO method to explore the role of B4galt1 in cerebral ischemic injury. As a result, B4galt1 may be regarded to be a possible substance in treating ischemic stroke.

BBB dysfunction is one of the hallmarks of ischemic stroke. The promotion of effective reperfusion and a favorable prognosis is a major concern for stroke patients. Astrocytes are considered to play a vital role in BBB function, angiogenesis and cerebral blood flow perfusion after ischemic stroke.^27^, ^28^ In our study, we found that rh‐B4galt1 reduced BBB extravasation and improved vascular reperfusion 24 h after ischemic stroke, but the exact mechanism remains unclear. The mechanisms by which B4galt1 participates in the progression of ischemic stroke need to be further investigated.

There is a growing interest in ferroptosis due to its important function.^29^, ^30^, ^31^ GPX4 is very important for regulating ferroptosis and SLC7A11 acts as an upstream mediator of GPX4. And ACSL4 works to be a biomarker and contributor of ferroptosis.^12^, ^32^ However, it remains unclear whether rh‐B4galt1 regulates cerebral ischemia‐induced ferroptosis. In this study, these levels of ROS, MDA, GSH, iron and several ferroptosis‐related key proteins, such as GPX4, SLC7A11 and ACSL4, were detected in mice possessing ischemic stroke. These results indicated that after cerebral ischemia, pro‐ferroptotic factors are upregulated, while anti‐ferroptotic factors are restrained, indicating the occurrence of ferroptosis. More importantly, our data showed that intranasal administration of rh‐B4galt1 could rescue I/R‐induced ferroptosis. We also confirmed that the iron chelator DFO ameliorated infarct volume and neurological deficit scores. All in all, we demonstrated that ferroptosis happens after cerebral ischemia and ferroptosis inhibition could alleviate cerebral ischemic injury. Therefore, we hypothesized that rh‐B4galt1 has a relationship with inhibition of ferroptosis, but particular mechanism should be ulteriorly explored.

Transcriptional co‐activator with PDZ‐binding motif (TAZ), one of the core modules of the Hippo pathway, is regulated by B4galt1 at the posttranslational level by acquiring N‐glycan modifications and B4galt1 could increase TAZ protein stability.^33^ After translocation into the nucleus, TAZ interacted with TEAD and bound to the promoter of Nrf2, whose blockage caused inability of TAZ to improve inflammation, implying that Nrf2 is a direct target of TAZ.^34^ We also determined that the rh‐B4galt1 had an important effect on TAZ/Nrf2/HO‐1 pathway. Its neuroprotective effects could be reversed by TAZ siRNA or Nrf2 inhibitor ML385, implying the important role of TAZ/Nrf2 in ferroptosis inhibition. In a word, rh‐B4galt1 came into play by suppressing ferroptosis in TAZ/Nrf2/HO‐1 pathway. However, the precise mechanism by which rh‐B4galt1 regulates TAZ in cerebral ischemic injury remains unclear and the molecular mechanism of rh‐B4galt1 against cerebral ischemic injury may involve multiple molecular pathways. The underlying mechanisms need to be further revealed before rh‐B4galt1 could be used for the treatment of ischemic stroke.

## 5 CONCLUSIONS

The results indicated that rh‐B4galt1 implements neuroprotective effects by modulating ferroptosis, primarily via upregulating TAZ/Nrf2/HO‐1 pathway. Rh‐B4galt1 was proved to have ability to prevent cerebral ischemic damage, suggesting its essential role in the treatment of ischemic stroke.

## AUTHOR CONTRIBUTIONS

Y.M. and C.L. performed the experiments and subsequent data analysis; L.R., J.L. and Y.X. helped perform experiments and review the manuscript. J.L., and P.W. designed the study and prepared the manuscript. All authors read and approved the final paper.

## FUNDING INFORMATION

This study was supported by the National Natural Science Foundation of China (no. 81971231), the Natural Science Foundation of Liaoning Province (no. 2022‐MS‐391), and the Scientific Research Project from the Educational Department of Liaoning Province (no. JYTMS20231733).

## CONFLICT OF INTEREST STATEMENT

The authors declare no competing interests.

## Supporting information


Supplementary Files 1.

## Data Availability

The datasets generated and/or analyzed during the current study are available from the corresponding author on reasonable request.
