# Supplementary material for: β‐1,4‐Galactosyltransferase 1 protects against cerebral ischemia injury in mice by suppressing ferroptosis via the TAZ/Nrf2/HO‐1 signaling pathway
Source: CNS Neurosci Ther. 2024 Sep 4;30(9):e70030. doi: 10.1111/cns.70030 (PMC11374693; doi:10.1111/cns.70030)

Full unedited gel/blot images

The full unedited Western Blots (WB) images are presented below, with the cropped image in the manuscript highlighted by orange rectangles.

Full unedited blot for Figure 1B

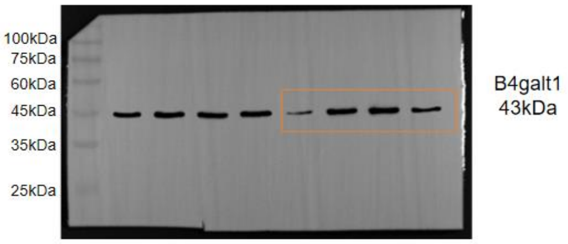

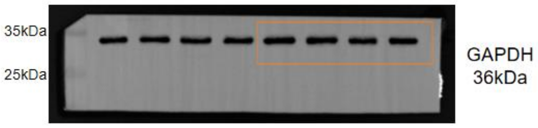

Full unedited blot for Figure 2A

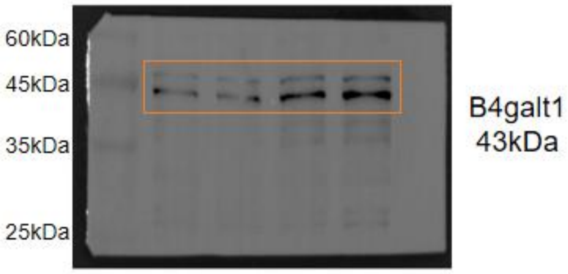

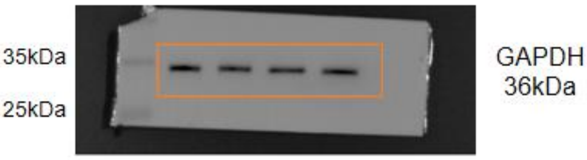

Full unedited blot for Figure 4E

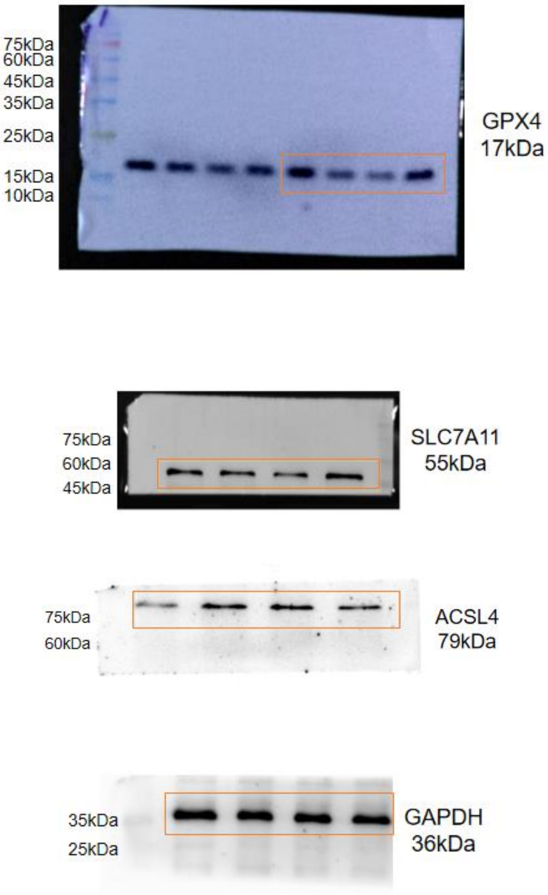

Full unedited blot for Figure 4J

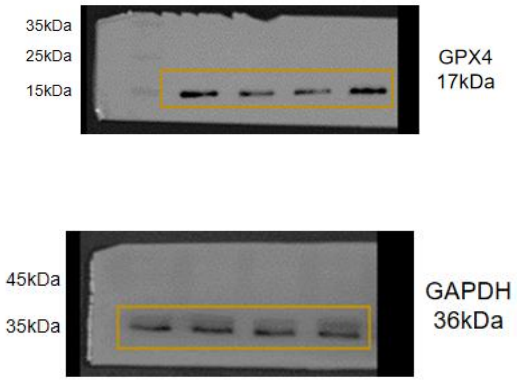

Full unedited blot for Figure 5A

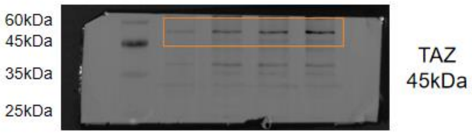

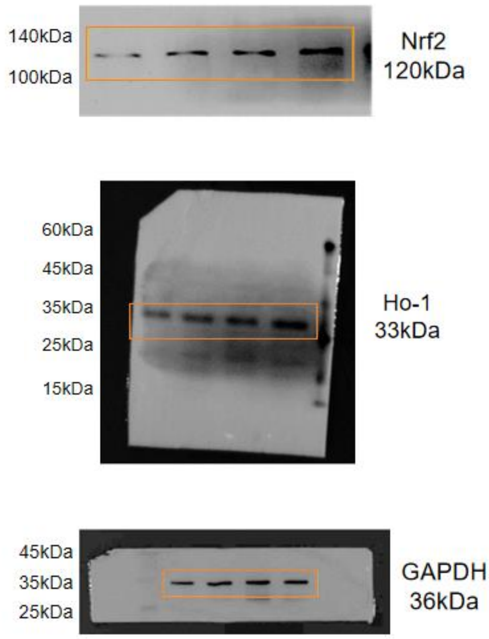

Full unedited blot for Figure 6A

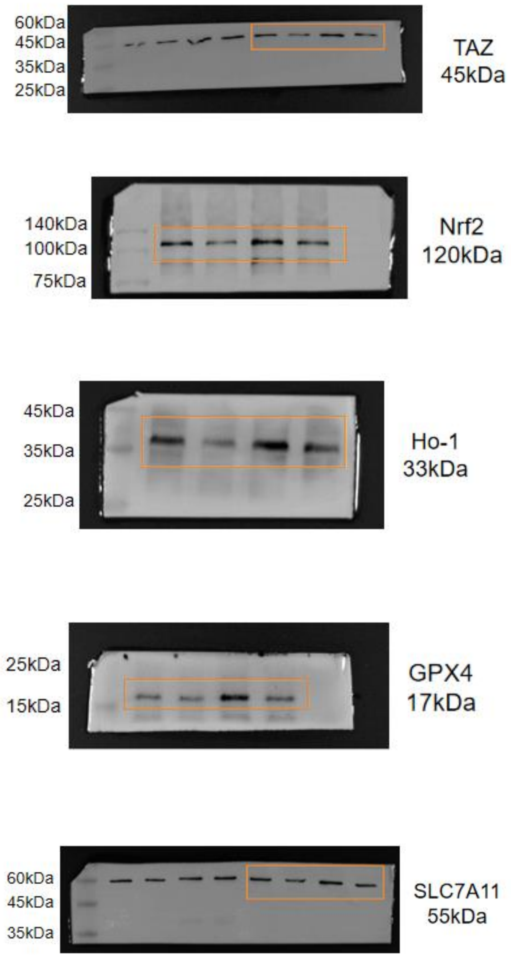

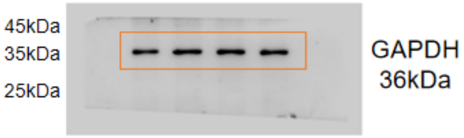

Full unedited blot for Figure 6H

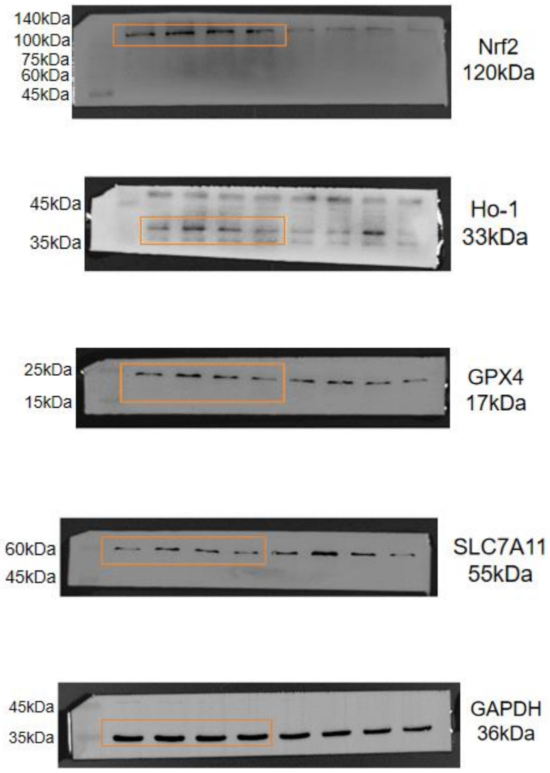

Supplement: Supplementary file 1 — Supplementary Files 1. [file CNS-30-e70030-s001.pdf]
